# Super Mario Bros. and Yoshi Games’ Affordance of Childlike Wonder and Reduced Burnout Risk in Young Adults: In-Depth Mixed Methods Cross-Sectional Study

**DOI:** 10.2196/84219

**Published:** 2025-12-19

**Authors:** Winze Tam, Congcong Hou, Andreas Benedikt Eisingerich

**Affiliations:** 1 Imperial College Business School Imperial College London London United Kingdom; 2 Faculty of Commerce Kyushu Sangyo University Fukuoka Japan

**Keywords:** childlike wonder, happiness, burnout, video games, qualitative research, survey study

## Abstract

**Background:**

Unrelenting pressure and an “always-on” culture can leave no time for genuine rest among young adults. While playing video games has been noted to afford cognitive escapism and relaxation, critical questions remain about the influence of popular video games, such as Super Mario Bros., and their potential effects on young adults’ burnout risk.

**Objective:**

This study examined the extent to which, if at all, popular video games such as Super Mario Bros. and Yoshi could foster childlike wonder. It also investigated the potential of these games to reduce burnout risk among young adults.

**Methods:**

We used a mixed methods approach. First, qualitative data were collected through 41 exploratory, in-depth interviews (women: n=19, 46.3%; men: n=21, 51.2%; prefer not to disclose sex: n=1, 2.4%; mean age 22.51, SD 1.52 years) with university students who had experience playing Super Mario Bros. or Yoshi. Second, quantitative data were collected in a cross-sectional survey (N=336) of players of Super Mario Bros. and Yoshi to examine the games’ affordance of childlike wonder, overall happiness in life, and burnout risk.

**Results:**

Insights from in-depth interviews showed that players appreciated the ability of Super Mario Bros. and Yoshi games to instill childlike wonder, enhance happiness in life, and reduce burnout risk. Quantitative analyses showed that the games’ affordance of childlike wonder positively affected young adults’ happiness (b=0.30, SE=0.04, t=6.80, 95% CI 0.21-0.38; *P*<.001). In turn, overall happiness significantly reduced the risk of burnout (b=–0.48, SE=0.05, t=–9.55, 95% CI –0.572 to –0.377). Results showed that happiness fully mediated the impact of childlike wonder on burnout, as the direct effect of childlike wonder on burnout risk became insignificant (b=–0.08, SE=0.04, t=–1.88, 95% CI –0.16 to 0.01; *P*=.06), while the indirect effect of childlike wonder on burnout risk was significant (b=–0.14, bootstrapped SE=0.03, 95% CI –0.20 to –0.09).

**Conclusions:**

The findings showed the significant positive effect of popular video games such as Super Mario Bros. and Yoshi on fostering players’ childlike wonder, increasing happiness, and reducing burnout risk. This study was among the first to identify childlike wonder as an emotional pathway through which mainstream video games could enhance well-being and reduce burnout. By moving beyond escapism and nostalgia, it offers a new perspective on how well-designed, globally familiar games can function as accessible, resilience-building digital microenvironments. These findings contributed to research bridging gaming and mental health and have practical implications for game designers, educators, and health professionals interested in promoting mental wellness through everyday play.

## Introduction

### Overview of Young Adults and Burnout Risk

Young people today face a unique constellation of interconnected pressures that contribute significantly to anxiety, worry, and burnout [1-3]. Soaring costs for housing, education (leading to significant student loan debt), and basic necessities create significant financial pressure [4,5]. Achieving traditional markers of adulthood (home ownership and a stable career) feels increasingly out of reach for numerous young people. Witnessing vast wealth disparities and feeling the system is rigged can fuel pessimism and a sense of unfairness about future prospects [6]. Furthermore, intense pressure to excel academically from a young age, secure prestigious internships or jobs, and build a seemingly perfect resume can create chronic stress [7]. Success is often narrowly defined and hypercompetitive. Importantly, these cumulative stressors arise during what developmental psychologists describe as emerging adulthood, a period marked by identity exploration and instability [8].

Young adulthood, often referred to as emerging adulthood, is considered a distinct stage of development characterized by identity exploration, instability, self-focus, and shifts in social roles, typically spanning the late teens and 20s [8]. Epidemiological research indicates that many mental disorders first appear during this life stage, although timing varies across diagnostic categories—for example, anxiety disorders tend to surface earlier, while mood disorders can appear later [9,10]. These onset patterns often coincide with major life transitions involving education, work, and growing autonomy, which have been linked to higher psychological distress and lower well-being among young adults [11]. Hence, focusing on this demographic remains critical for understanding today’s mental health challenges and for identifying where digital interventions might best support well-being.

In addition, the current “always-on” digital culture and social media expose young people to curated highlights of others’ lives, careers, and achievements 24/7, fostering unrealistic comparisons, fear of missing out, and feelings of inadequacy [12-15]. The digital world also provides new avenues for bullying and harassment, impacting mental health significantly. Moreover, smartphones make it difficult to disconnect from work, studies, or social obligations [16,17]. The expectation to be constantly available and responsive erodes downtime and contributes to burnout. Moreover, doomscrolling or the constant exposure to negative news cycles and global crises (climate change, political or social conflict and unrest, or pandemics) can be overwhelming and induce feelings of helplessness [18-20]. Fueled by social media and competitive environments, many young adults internalize unrealistic expectations of perfection in all areas (looks, career, relationships, and hobbies), leading to self-criticism and anxiety about failure [21-23].

Moreover, while digitally connected, many young adults report feeling lonelier than ever [6,12,24]. Traditional community structures (extended family, local community groups, and religious institutions) often play a less central role nowadays. Despite online networks, forming and maintaining deep, meaningful in-person relationships can be challenging in what is often perceived as a fast-paced, transient world [6]. Taken together, these factors can create a state of chronic, unrelenting stress [6,25-27]. Feeling powerless over economic realities, global issues, or demanding work or school environments fuels burnout. Constant pressure and the “always-on” culture leave little time for genuine rest, relaxation, and hobbies that replenish energy. Hence, these factors do not exist in isolation. They feed into each other.

Social media comparisons fuel anxiety and worry about the future sap motivation, while perceived loneliness makes coping harder. It creates a vicious cycle that can be difficult to break. This study aims to make a step toward addressing the mental health crisis facing many young adults today. More specifically, video games, such as open-world games, have been shown in prior work to afford cognitive escapism, relaxation, and enhanced well-being to young people [28]. Video games and gamification techniques have been noted to positively affect the well-being of players in different ways [29-34]. However, critical questions remain about the potential role popular video games can play in young adults’ lives.

### Background on Super Mario Bros. and Yoshi Games

The Super Mario Bros. franchise created by Nintendo Co, Ltd stands as one of the most globally popular and recognizable in entertainment history [35,36]. Since the groundbreaking original in 1985, Mario games have sold more than 430 million copies worldwide, making it one of the best-selling video game series ever. Its appeal transcends age, culture, and nationality. Super Mario, Luigi, Princess Peach, and the cute dinosaur Yoshi are all cultural icons, instantly recognizable even to nongamers. Super Mario Bros. and Yoshi games can help act as stress buffers and reduce negative affect in several critical ways. For example, engaging with the demanding spatial navigation, timing, and problem-solving tasks in Super Mario Bros. requires focused attention (working memory load). This acts as a distractor, effectively diverting finite cognitive resources away from rumination on real-world worries or negative thoughts, thereby interrupting cycles of pessimism or anxiety. Players become deeply immersed in “being” Mario or Yoshi, navigating the space, temporarily suspending self-referential thoughts and worries about real-life identity or performance.

Furthermore, the core narrative of Mario and Yoshi persistently overcoming obstacles to achieve goals serves as a powerful symbolic representation of perseverance and eventual triumph. Engaging with this narrative, especially through active participation (the player is Mario or Yoshi), can subtly reinforce schemas related to resilience and positive outcomes. The core objective (“Help Prince Florian” or “Rescue Princess Peach”) is unambiguous and morally positive, offering a clear purpose without complex emotional baggage or ambiguity. This reduces cognitive load associated with real-world problem interpretation. While challenging in their own ways, Super Mario Bros. and Yoshi games generally lack the prolonged high-stress tension of survival horror or competitive multiplayer games. Their rhythm often involves bursts of focused action followed by brief moments of lower tension (eg, traversing cleared sections), allowing for a more sustainable and restorative form of play.

Thus, Super Mario Bros. and Yoshi games can be a potent tool for temporary stress reduction, mood enhancement, and cognitive restoration, creating a mental “reset” that can foster a more optimistic and resourceful return to daily life. Their specific game design, combining skill demands with low real-world stakes, predictable rules, and positive affect induction, can make these games an effective vehicle for enhanced mental well-being. Super Mario Bros. and Yoshi games, hence, can act as an antidote by creating a virtual space that is the psychological inverse of a burnout-inducing environment.

### Enhancing Childlike Wonder and Reducing Burnout Risk

Super Mario Bros. and Yoshi video games can offer pure, unadulterated joy and playfulness. More specifically, Super Mario Bros. games may help rekindle childlike wonder and help players see magic in the everyday. Jumping, running, breaking blocks, and collecting coins; these actions are inherently joyful and tactile. The satisfying “boing” of a jump, the “ching” of a coin, the smash of a brick; all these can trigger simple, primal pleasure. Mushrooms make players big, flowers shoot fire, stars make players invincible, turtles are cute enemies, clouds and trees have eyes! It presents a world operating on its own delightful, nonsensical logic, bypassing adult cynicism.

In addition, the vibrant colors, upbeat music, and generally sunny atmosphere (even underground or in castles has a playful menace) create an optimistic baseline. It is hard to stay cynical when that iconic tune is playing. In essence, Super Mario Bros. games act like a playful lens. It reminds players of the joy of pure discovery, the thrill of simple challenges, the magic of transformation, and the power of imagination that they possessed as children.

Furthermore, the visual and auditory design of Yoshi games is meticulously crafted to feel safe, warm, and inviting. Games such as *Yoshi’s Crafted World* and *Yoshi’s Woolly World* use a tangible, diorama-like aesthetic. Everything appears to be made of yarn, cardboard, and craft supplies. This evokes a sense of handmade charm and nostalgia, reminiscent of childhood arts and crafts. The world feels nonthreatening and playful. Yoshi games are bursting with joyful, bright colors, while the music is famously cheerful and melodic. Yoshi’s vocalizations (the flutter jump and the egg spit) and the sounds of collectibles are satisfying and cute. This creates a consistent visual and audio landscape of positivity.

Burnout is characterized by exhaustion, cynicism (detachment or negativity), and reduced efficacy. Super Mario Bros. and Yoshi video gameplay can help reduce burnout risk in the following ways. First, the attentional demand of Super Mario Bros. and Yoshi gameplay provides a mental break from work or role-related rumination (a core driver of exhaustion). This allows depleted cognitive resources (eg, prefrontal cortex function taxed by decision fatigue) to replenish. Induced flow states and positive affect (dopamine or serotonin release) counter chronic stress responses (eg, lowered cortisol and reduced sympathetic nervous system activation). This provides physiological recovery crucial for combating exhaustion.

Furthermore, Super Mario Bros. and Yoshi games’ rewarding mechanics and light-hearted aesthetic actively elevate mood, counteracting the persistent negative affect associated with burnout. That is, the game’s core loop reinforces the belief that effort leads to reward. Collecting coins, finding secrets, or beating a level after multiple tries demonstrates a reliable, predictable link between action and positive outcome, rebuilding trust in effort that burnout erodes. Moreover, the games’ narrative (perseverance leading to success) and aesthetics (colorful and playful) provide exposure to positive, noncynical representations, subtly challenging pervasive negative worldviews fostered by burnout.

Moreover, Yoshi games are also famously accessible. This removes the stress of failure and allows players to simply enjoy being in the world. The challenge comes from discovery and 100% completion, not from mere survival, making it a choice rather than a demand. While burnout is often fueled by anxiety about the future or rumination on the past, Yoshi games anchor players in the now.

Taken together, Super Mario Bros. and Yoshi games provide psychological restoration. The warm aesthetics, cheerful music, and a lack of intense pressure are not draining. They are replenishing. Playing feels like a break for the brain, similar to the effect of a walk in a pleasant park. The charm and kindness inherent in these games make it hard to be cynical. The games offer joyful sounds and enchanting visual feedback that help rebuild a sense of competence and agency in a low-pressure environment, countering the helplessness that fuels burnout.

Thus, in this study we aim to investigate if popular video games such as Nintendo’s classic Super Mario Bros. and Yoshi franchise are able to promote players’ childlike wonder—a state of openness, curiosity, and delight in the face of the new or mundane—and whether such experiences enhance overall happiness in life and reduce the risk of burnout among young adults.

## Methods

### Study 1

#### Research Design Overview

The findings of our qualitative study were reported in accordance with the Standards for Reporting Qualitative Research guidelines [37]. In addition, we adhered to the Journal Article Reporting Standards-mixed methods article reporting standards and offered information recommended for inclusion in research that reports the collection and integration of qualitative and quantitative data [38]. We conducted semistructured, cross-sectional interviews with open-ended, predetermined questions to explore and consider different personal opinions and views and help generate novel insights from data gathered in a natural setting [39,40]. Specifically, we invited study participants to share their thoughts about Super Mario Bros. and Yoshi games, and how, if at all, such a video game affects their day-to-day life. That is, during the interview, study participants were given an opportunity to reflect on and discuss how Super Mario Bros. and Yoshi games made them feel, what benefits (if any) they got from playing the game, and how and why they played the game. In case study participants played more than one Super Mario Bros. and Yoshi game, they were invited to reflect on the game they played the most.

#### Researcher Description

To help minimize any potential bias, 4 trained research assistants (RAs) conducted the interviews. RAs were trained and instructed not to guide the interviewees and their responses in any way. The team of RAs was diverse in sex (2 women and 2 men) and ethnicity (from 3 different continents) to help ensure increased comfort with study participants during interviews.

#### Study Participants or Data Sources

##### Participant Recruitment

As part of our qualitative study, we used a convenience sampling method. Potential participants were recruited via posters and by handing out flyers to students on a university campus in July and August 2025.

##### Participant Selection

The specific eligibility criteria for taking part in the qualitative study were that participants were full-time students and that they had some personal experience playing a Super Mario Bros. or Yoshi game before.

##### Data Collection

Over the course of 3 weeks, exploratory, in-depth interviews were conducted with 41 participants (women: n=19, 46.3%; men: n=21, 51.2%; preferred not to disclose sex: n=1, 2.4%; mean age 22.51, SD 1.52 years). All interviewees were full-time students (confirmed by their student IDs) and had experience playing a Super Mario Bros. or Yoshi game (screening questions by the RAs included the name of the specific Super Mario Bros. or Yoshi games respondents had played and which console they used to play the game, eg, Wii U, Nintendo Switch, or Nintendo Switch 2). Interviews were conducted in a university cafeteria and lasted between 25 and 40 minutes. All interviewees were reassured of the anonymity of their responses and were informed that their participation would help inform academic research. At the end of the interview, each interviewee was given a chance to ask any questions they may have had.

##### Statistical Analysis

We examined our qualitative data gathered from the interviews, following recommended and established procedures for qualitative data analyses [40-42]. RAs had a chance to familiarize themselves with the data collected by transcribing the interviews and carefully reading the transcripts. Furthermore, the coding of interview data was conducted in duplicate by the authors, and any potential discrepancy was discussed and resolved. In doing so, we followed the constant comparison technique in prior published work, collected and analyzed interview data in tandem, and continuously compared the newly collected data against the existing insights. As a first step of our analysis, we grouped conceptually linked data and reduced them to a set of meaningful concepts. That is, we examined and analyzed the data to develop insights and a holistic understanding of interviewees’ responses and key elements of Super Mario Bros. or Yoshi games that were noted to affect their lives.

In addition, we used descriptive phrases and open coding to develop further insights regarding key drivers and potential process explanations about interviewees’ reflections of Super Mario Bros. or Yoshi games and critical elements of gameplay that may influence their overall happiness in life and burnout risk. Moreover, in joint discussions by the team of RAs and authors, we integrated relevant literature in an iterative process, further explored key elements of Super Mario Bros. or Yoshi games that influenced players’ lives, and finalized the key elements of our conceptual framework.

### Study 2

#### Research Design

As part of study 2, we conducted a cross-sectional survey using a convenience sampling approach.

#### Inclusion and Exclusion

Participants were deemed eligible to take part in the study if they were full-time university students at the time of completing the study and if they had personal experience playing a Super Mario Bros. or Yoshi game. We conducted the study in July and August 2025 and followed the Strengthening the Reporting of Observational Studies in Epidemiology (STROBE) guidelines to report the observational, cross-sectional studies (Multimedia Appendix 1) [43].

#### Sampling Procedures

Potential study participants were recruited by handing out flyers and using posters on a university campus in July and August 2025. Trained RAs informed potential study participants that the study would take no longer than 15 minutes and that each participant would receive US $5 (approximately £4) as compensation for taking part in the study.

#### Sample Size, Power, and Precision

Over the course of 5 weeks, 4 RAs helped with the data collection (student ID check and simple screening questions before the study, ie, which Super Mario Bros. or Yoshi game participants had played before and which console they had used for gameplay, such as Wii U, Nintendo Switch, Nintendo Switch 2, etc) and managed to recruit a total of 350 participants. Because 14 participants did not complete the entire survey, their responses were discarded from the study, and the effective number of responses was 336. In case study participants noted playing more than one Super Mario Bros. or Yoshi game, they were encouraged to consider and think of the game they played the most when answering the study’s survey questions. RAs invited participants to complete a brief paper-and-pencil questionnaire about gaming and their daily life activities.

#### Participant Characteristics

The final sample consisted of 336 full-time university students (women: 19/41, 46.3%; men: 21/41, 51.2%; prefer not to disclose sex: 1/41, 2.4%; mean age 22.51, SD 1.52).

#### Measures and Covariates

Specifically, participants answered questions about burnout risk that were adapted from validated, previously published scales designed to measure burnout risk [44,45], and included items such as “I feel emotionally drained by my daily responsibilities (caregiving, work, etc),” “I have become more cynical or detached about activities I used to care about,” and “I doubt whether my efforts actually make a difference”).

In addition, participants were invited to answer questions to measure their overall happiness in life. Measurement items to capture life happiness were adapted from previously published happiness in life scales [46,47] and consisted of 3 items (“I feel grateful for the good things in my life,” “I am satisfied with the overall direction of my life and look forward to what lies ahead,” “Overall I would describe myself as a happy person”). Moreover, we captured the extent to which gameplay enhanced participants’ childlike wonder by adapting three items from previously published scale items on noticing novelty and joy in discovery, feeling wonder in response to stimuli [48-50] (“Playing a Super Mario Bros./Yoshi game helps me feel delighted by small, ordinary things that others overlook (e.g., shapes in clouds, patterns in pavement),” “Super Mario Bros./Yoshi gameplay encourages me stop to admire or explore things that spark my curiosity, even if they seem unimportant (e.g., a hidden path, an unusual insect),” “Playing a Super Mario Bros./Yoshi game encourages me to pause and to appreciate playful or whimsical details in my environment”). All responses were measured on a 7-point Likert scale (1=strongly disagree, 7=strongly agree). As control, participants indicated their sex (1=female, 2=male, 3=prefer not to disclose).

To ensure the quality of measurement, all scale items were reviewed by the research team for clarity and consistency before survey administration. The questionnaire was distributed in printed form on campus and completed individually by respondents. RAs who facilitated recruitment and survey distribution were not informed of the study’s hypotheses and followed standardized instructions to minimize expectancy or response bias. Psychometric properties of the measures were examined using confirmatory factor analyses and reliability testing. All factor loadings were significant (*P*<.001). Cronbach α, composite reliability (CR), and average variance extracted (AVE) all exceeded widely accepted thresholds (α ≥.70, CR ≥0.70, and AVE ≥0.50), indicating strong internal consistency and convergent validity [51-53]. The Fornell-Larcker criterion further supported discriminant validity across constructs [53].

#### Statistical Analysis

We followed recommended procedures for conducting confirmatory factor analyses and examined the factor structure and reliability scores for the measurement items in our survey study [51,52]. This was done to examine whether the items significantly loaded on their intended factors and had weak cross-loadings. Furthermore, we examined the AVE and CR scores for the scales used in our study. Moreover, we tested whether the average variance extracted for each construct was greater than the squared correlations between the construct and other variables in our survey [53]. The hypothesized 3-factor model (childlike wonder, happiness, and burnout risk) was tested using confirmatory factor analysis in IBM SPSS AMOS 29.0 with maximum likelihood estimation.

In addition, we conducted a mediation analysis with the PROCESS macro [54] (model 4; bootstrapped samples=5000; 95% CI) to examine the influence of Super Mario Bros. and Yoshi games’ affordance of childlike wonder (independent variable) on overall happiness in life (mediator) and, consequently, on players’ burnout risk (dependent variable). In order for PROCESS not to treat sex as a continuous variable, sex was coded as a dummy variable (0=female, 1=male). Due to the insufficient sample size (n=1), the “prefer not to disclose” category was excluded from analysis.

### Ethical Considerations

#### Study 1

Ethics approval for the study was received from Imperial College London’s Analytics, Marketing, and Operations (AMO) Department. At the time of data collection, the AMO Department followed Imperial College London’s ethics protocol for research involving human participants, which includes internal review and written approval by the departmental head of the research group. The procedure ensures compliance with Imperial College Research Ethics Committee guidelines and the principles of the Declaration of Helsinki. Informed consent was obtained from all participants, and participants were informed that they had the right to withdraw from the study at any time point. Study data were anonymized and kept confidential within the research team. More specifically, we ensured that no identification of individual study participants in any images or supplementary material of the manuscript was possible. After the interview was completed, each participant was thanked, debriefed about the study’s purpose, and given £15 (approximately US $20) as compensation for their time.

#### Study 2

For study 2, ethics approval was received from Imperial College London at the Departmental level, Analytics, Marketing, and Operations. The AMO Department follows Imperial College London’s ethics protocol for research involving human participants, which includes internal review and written approval by the departmental head of the research group. This procedure ensures compliance with the Imperial College Research Ethics Committee guidelines and the principles of the Declaration of Helsinki. All data collected were anonymized and kept confidential within the research team. Study participants provided informed consent and were informed that they had the right to withdraw from the study at any time and without having to give a particular reason. We ensured that no identification of individual study participants in any supplementary material or in any images of the manuscript was possible. After completing the survey, each participant was thanked, debriefed, received £4 (approximately US $5) as compensation for their time, and was invited to ask any questions they may have had about the study.

## Results

### Study 1: Overview

The findings of the in-depth interviews demonstrated that Super Mario Bros. and Yoshi games’ affordance of childlike wonder played an important role in their impact on players’ lives. In Table 1, we list the representative quotes, which indicate the extent to which players viewed Super Mario Bros. and Yoshi games as fostering childlike wonder and the influence they had on their day-to-day life. In addition, interviewees noted how Super Mario Bros. and Yoshi gameplay affected their overall happiness in life as well as how gameplay helped reduce their risk of burnout (refer to Table 1 for representative quotes).

**Figure 1:**
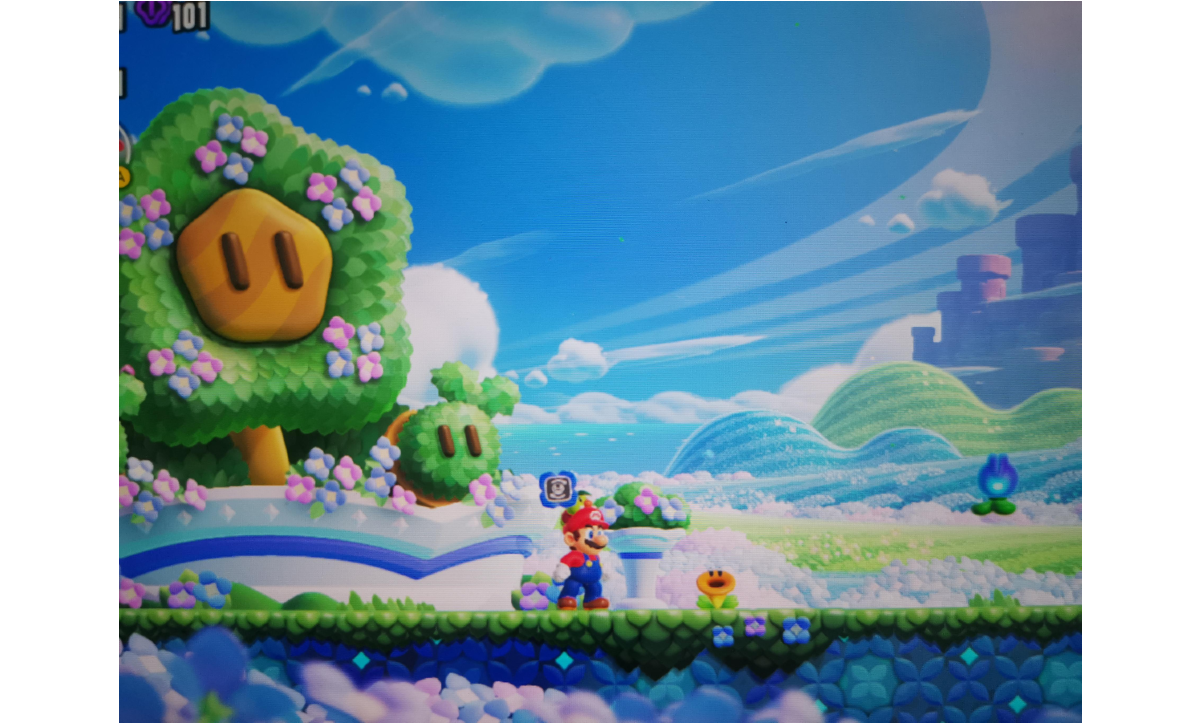
Cheerful landscape in *Super Mario Bros. Wonder* (reproduced with permission from Nintendo).

Participants frequently described the bright colors and optimistic tone of *Super Mario Bros. Wonder* as evoking feelings of calm and childlike joy. Figure 1 illustrates this visual atmosphere, depicting the cheerful landscape that several interviewees referred to when recalling moments of wonder and relaxation.

**Figure 2:**
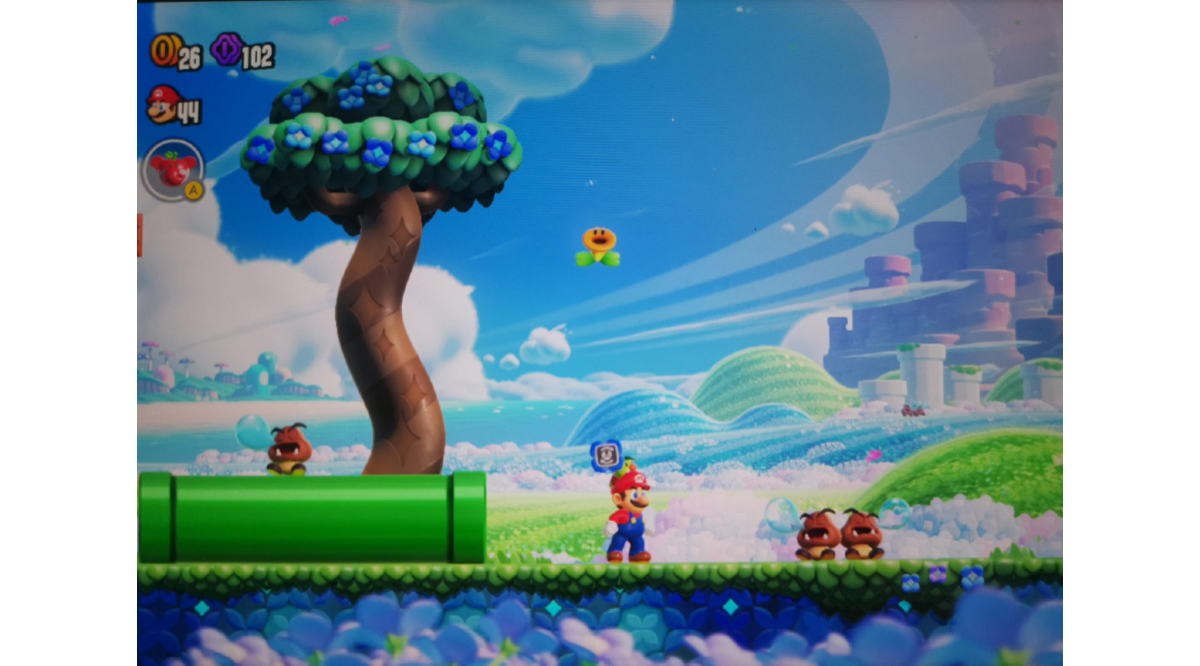
Super Mario facing sleeping enemies in *Super Mario Bros. Wonder* (reproduced with permission from Nintendo).

Interviewees highlighted how the game’s design balances challenge with playfulness, creating a sense of ease that contrasts with daily pressures. Figure 2 exemplifies this tone, showing even “enemies” portrayed in a gentle, nonthreatening way—an aspect the interviewees associated with relaxation and stress relief.

**Figure 3:**
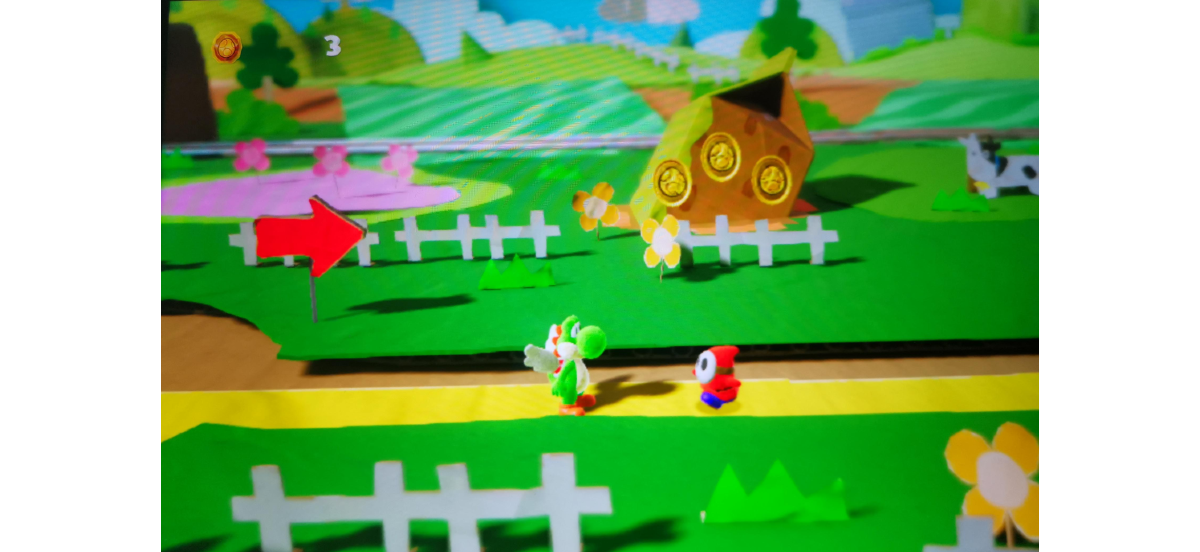
The bright and cheerful environment of *Yoshi’s Crafted World* (reproduced with permission from Nintendo).

Interviewees often described *Yoshi’s Crafted World* as a warm, inviting environment that felt comforting and safe. Figure 3 illustrates the handcrafted, diorama-like aesthetic that many associated with nostalgia and emotional restoration.

**Table 1:** Representative quotes of interviewees in an in-depth qualitative study about the key effects of Super Mario Bros. and Yoshi games and their influence on their lives.

Game key effects	Representative quotes
Childlike wonder	“Playing Yoshi transports me back in time. It’s a bit like time travel. I see myself as this kid again, wondering about the possibilities and beauty of this world. It’s a bit like a fresh new start.” [Respondent 8] “Super Mario Bros. Wonder is crazy. I love how the clouds are all animated and full of life. I hardly see the sky these days. Living in a big city it’s hard to get a sense of nature. Deep down that certainly must affect people. We did not evolve over thousands of years to run around like rats underground, squashed in hot and cramped, tight spaces like the subway and compete for the tiniest of spaces. Super Mario Bros. Wonder is like a breath of fresh air with trees, clouds, water and fun everywhere. It makes me feel I am part of nature and nature is part of me. I miss that feeling. When I play Super Mario Wonder it is as if I’m a kid again.” [Respondent 13] “Haha, the moment I switch on and the game starts with the iconic Super Mario tune in my head I sense a moment of relief and I am playing the game as I used to when I was a child. Probably that was the happiest time of my life when I was a child. Super Mario gives me a chance to appreciate the world and everything again with the eyes of a child that is not jaded, not tired but full of energy, full of enthusiasm, interest, and marveling at what surrounds us and what each new day may bring.” [Respondent 16] “Playing with friends or just by myself lying on the bed, Super Mario Bros. games make me feel warm and fuzzy and comfortable. A bit like being wrapped in a cozy, warm blanket on a cold winter day when it is snowing outside and as a kid you are dreaming about the snowflakes and wondering how much snow may fall today. Most days I feel exhausted and no energy to appreciate things anymore. Playing Super Mario gives me this new sense of being a kid with wide, interested eyes wanting to learn and explore again.” [Respondent 30] “There is nothing too fancy about Super Mario games. Come to think about it, that’s what probably makes the Super Mario Bros. games so special. They hold your hand and let you find that first super mushroom, make that first jump, all in a way that feels like play. It is not a chore and does not feel like a nuisance but it’s a game! The colors are so vibrant and fun. The music and tunes are cheerful and put a smile on my face every single time. I play and I cannot help but wonder about… small things. There is this fly on my window. But I don’t mind. Maybe the fly will find a way out. I hear the rain outside and I keep looking at the clouds. I think of the clouds in the game and smile. The game makes me feel like a kid in a candy store. You keep looking at all the sweets with amazement, just wondering which one of these incredible candies should be the one to be picked? What a treat!” [Respondent 39] It is easy to dismiss this but then I feel it is true. Playing Yoshi games makes me feel like… hey, don’t take everything so seriously out there. You see, it is easy to get lost in all sorts of worries and anxieties, stress and negativity. I don’t see the flowers, the sky, the clouds, the sunset or the sunrise. I have become blind to the beauty of well everything really. But Yoshi video games open my eyes again to that way of looking at the world like a kid in a candy store. Running full speed on top of the clouds? … Hahaha, let’s a go!” [Respondent 40]
Overall happiness in life	“Look at your phone and everyone tells you to buy more, to chase the big dream and exotic destinations and you will get happy there. Only to keep chasing, keep buying more, travel to no end to get lost, and all the while happiness may be just around the corner. Super Mario games give me that feeling that happiness is truly in the small things in life. It does not take a trip to the Maldives. What are you chasing? Stop chasing, it’s right there in front of your eyes. You don’t have to spend millions to feel happy. Do you know what’s crazy? Playing my Super Mario on the Nintendo Switch makes me think back of the time when I played Super Mario waiting in the dentist’s waiting room. And you go, hold on a minute, the dentist room?!? That’s the least likely place to feel happiness and you are right. But I still remember, I am sitting there and I completed the game. I completed the game and listened to the credits roll on and that iconic theme… that music… at that moment, I felt happiness. This is the pure magic of playing Super Mario. You can be sitting in a dentist’s waiting room and feel happiness. That’s truly awesome. Super Mario is simple, pure, happiness.” [Respondent 2] “So I often think about what it means to be happy. There are zillion of books on this and the internet has even more answers. Everyone seems to know what will definitely make you happy. You need a house. You need to run that marathon. You need to look like this and buy that. When was the last time I felt truly happy. I mean truly happy? It’s when I played Super Mario Bros. Wonder and I switched on and was there in the moment. When I jumped, I jumped, and when I ran, I ran. That was happiness.” [Respondent 17] “The most precious resources we may have are time, health, knowledge and skill, family and the most previous things in life are free like health. Social media tells me I need to dream big and do big things to achieve big things, everything has to be crazy big. Happiness may be in small things, in the quiet moments, in the time when I am allowed to be myself and just be there and appreciate the moment I have. Super Mario is this simple happiness that made me feel so… happy, there is no other word for it, when I was a kid. Happiness as a kid did not have to be complicated. Happiness was completing a level. Happiness was spending the summer holidays doing nothing special or grand but just enjoying the life one had.” [Respondent 25] “I love level one in my Super Mario Bros. U Deluxe. The grass is green and the clouds and the sky look so cheerful and endearing. It makes me feel I want to walk through the woods, climb trees, balance, and jump off. Or maybe I should lie on the grass and watch the clouds fly by high in the sky waiting for rain to come or a summer thunderstorm or even rolling thunder and snow? Super Mario Bros. U Deluxe reminds me that I am part of nature and it’s here for me. Jumping, climbing, jumping, picking up a leaf, a flower. Nature is not just an amazing backdrop in Super Mario, it’s integral to the game and how it makes me smile again.” [Respondent 26] “I kept jumping and falling. Jumping and falling all the time. Finally I made the jump and I was over the moon. What a feeling of happiness. I still remember it. That Super Mario theme is pure, catchy video game magic and happiness for the tired soul! Cannot feel not happy when listening to it. It’s not a big, dramatic piece. Super Mario games are like that. Happiness in the simplest and clean form.” [Respondent 33] “A while ago I did have the strangest of feelings. I was sitting on my sofa and played Super Mario when I looked outside the window and spotted the moon shining. I haven’t seen the moon for ages. I mean ages. And there it was shining and smiling at me. At least I felt it was smiling at me. I even discovered a few stars. Super Mario is a magical treat for the soul. The game does not shout but it gently holds you and lets you explore by yourself. Why would playing a video game make me appreciate my natural surroundings more? It is not obvious to me. Probably on the subconscious level more than anything Super Mario allows me to be part of nature how humans did for centuries, running around, trying to get a lucky break and find a leaf, flower, mushroom that gives them some much needed energy boost, jumping, exploring, avoiding danger, and finally making it back home.” [Respondent 34] “Keep collecting those coins of joy. Power-up with little moments of happiness and never stop looking for hidden blocks 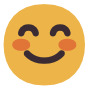 . That’s what Super Mario taught me more than anything.” [Respondent 37]
Burnout risk reduction	“Yoshi games help me see the world and my life with softness, playfulness and wonder. The fact that it allows me to appreciate the magic in something as simple and joyful as Super Mario is what brings me joy. Never underestimate the power of play. Life can feel meaningless and empty. Whether it’s jumping as Yoshi or noticing the way light hits a glass window, you are actively co-creating a kinder, more magical world just by choosing to see it that way. This may be Yoshi’s biggest super power.” [Respondent 8] “Sad how cynical life has become. Do you know when it all started? What is the cause of this? Everyone is cynical and tired of life. It’s almost as if humans have given up already. Tragic really when to think about it. When I play my Nintendo Yoshi games I feel less anxious and worry and the crises in my life seem that little bit further away. That moment of inner peace is priceless.” [Respondent 11] “It is hard to feel excited and energized how I used to. Life just keeps happening to you. It’s everywhere you look though. Yoshi, Princess Peach, Super Mario and the whole gang brighten my days just keep on running and jumping up that flagpole as high as you can. It gives me a bit more energy and less worry, a bit more strength to keep going another day and not keep thinking about all the things.” [Respondent 28] “Burnout may be the shadow of deep care. If you feel its weight. Breathe. Remember you don’t need to carry the wonder. You are the wonder. Mario is our fellow traveler in this journey. Super Mario games allow me to see the magic in small things. It’s a true gift to feel life has still something in store and not being tired of it all.” [Respondent 33] “Do you sometimes feel tired of it all? I do. What helps me is to step out and do things that give me space to stop worrying and overthinking things. For example when I play my favorite Yoshi on my Nintendo Switch and get some energy back and motivation back in life. Not everything seems lost anymore when you got Yoshi to cheer you on.” [Respondent 32] “Weird how pointless life can be or feel that way. The news gives me thousand things happening at the same time. So much violence, war, hate, abuse, disconnection, all the time. I need a break from this. It just makes me feel exhausted and I cannot make a difference anyway. I am not going to stop that war or people from acting selfishly. I feel lost and sad. Super Mario gives me back some happiness and moment of when I can enjoy the time so I do not lose hope entirely or willingness to do anything. It’s a happiness booster that helps me keep going a bit longer and keep hanging in there. It’s nice to feel life still has some happy moments to offer and not just trouble. To level up and keep looking forward to the next adventure.” [Respondent 41]

### Study 2: Statistical Analysis Results

Detailed survey measurement items and reliability scores are listed in Table 2. Confirmatory factor analyses showed that all measurement items significantly loaded on their intended factors (>0.79; *P*<.001) with weak cross-loadings (<0.27). In addition, AVE and CR scores exceeded the recommended thresholds. In addition, the Cronbach α scores were generally high and results showed that the AVE for each construct was greater than the squared correlations between the construct and other variables in our survey in support of discriminant validity (Table 2). The model demonstrated good fit to the data (*χ*^2^_24_=30.6, Tucker-Lewis index=0.99, comparative fit index=0.99, normed fit index=0.98, and root mean square error of approximation=0.03; 90% CI 0.00-0.06; *P*=.17). Estimation of standardized residuals showed that no values were particularly large (>2.00), indicating no substantial localized areas of misfit.

**Table 2:** Cross-sectional survey measurement items’ factor loadings and reliabilities.

Survey study constructs and measurement items			Factor loadings
**Burnout risk^a^**			
	I feel emotionally drained by my daily responsibilities (caregiving, work, etc)	.84	
	I have become more cynical or detached about activities I used to care about	.79	
	I doubt whether my efforts actually make a difference	.87	
**Happiness in life^b^**			
	I feel grateful for the good things in my life	.85	
	I am satisfied with the overall direction of my life and look forward to what lies ahead	.80	
	Overall I would describe myself as a happy person	.87	
**Childlike wonder^c^**			
	Playing a Super Mario Bros/Yoshi game helps me feel delighted by small, ordinary things that others overlook (eg, shapes in clouds, patterns in pavement)	.92	
	Playing a Super Mario Bros/Yoshi game encourages me to pause and to appreciate playful or whimsical details in my environment	.90	
	Super Mario Bros/Yoshi gameplay encourages me to stop to admire or explore things that spark my curiosity, even if they seem unimportant (eg, a hidden path, an unusual insect)	.91	

^a^α=.83; composite reliability (CR)=0.87; average variance extracted (AVE)=0.70.

^b^α=.86; CR=0.88; AVE=0.71.

^c^α=.92; CR=0.94; AVE=0.83.

### Study 2: Mediation Analysis Results

The results of the mediation analysis with PROCESS macro [54] (model 4; bootstrapped samples=5000; 95% CI; sample size=336) showed that Super Mario Bros. and Yoshi games’ affordance of childlike wonder (independent variable) had a significant, positive influence on players’ overall happiness in life (b=0.30, SE=0.04, *t*=6.8, 95% CI 0.21-0.38; *P*<.001; Table 3). In turn, overall happiness in life significantly reduced players’ burnout risk (b=–0.48, SE=0.05, *t*=–9.55, 95% CI –0.57 to –0.38; Table 4). Gender had no significant effect on happiness (b=–0.05, SE=0.12, *t*=–0.42, 95% CI –0.29 to 0.19; *P*=.68; Table 3) and no significant effect on burnout (b=0.02, SE=0.11, *t*=0.14, 95% CI –0.20 to 0.23; *P*=.89; Table 4). Furthermore, the results showed that happiness fully mediated the impact of childlike wonder on burnout, as the direct effect of childlike wonder on burnout risk became insignificant (b=–0.08, SE=0.04, *t*=–1.88, 95% CI –0.16 to 0.01; Table 4), while the indirect effect of childlike wonder on burnout risk was significant (b=–0.14, BootSE=0.03, 95% CI –0.20 to –0.09).

**Table 3:** Cross-sectional survey mediation regression results: effect of childlike wonder on happiness.

Predictor		b (95% CI)	SE		*t* test		*P* value
**DV^a^: Happiness^b^**							
	Constant	2.50 (2.07 to 2.93)	0.22	11.43		<.001	
	Childlike wonder	0.30 (0.21 to 0.38)	0.04	6.79		<.001	
	Gender	–0.05 (–0.29 to 0.19)	0.12	–0.42		.68	

^a^DV: dependent variable.

^b^*R*=0.35, *R*²=0.12, mean squared error=1.43, *F*_2, 333_=23.16; *P*<.001.

**Table 4:** Cross-sectional survey mediation regression results: effect of happiness on burnout.

Predictor		b (95% CI)	SE	*t* test	*P* value
**DV^a^: Burnout^b^**					
	Constant	5.37 (4.91-5.83)	0.23	22.99	<.001
	Childlike wonder	–0.08 (–0.16 to 0.00)	0.04	–1.88	.06
	Happiness	–0.48 (–0.57 to –0.38)	0.05	–9.56	<.001
	Gender	0.02 (–0.20 to 0.23)	0.11	0.14	.89

^a^DV: dependent variable.

^b^*R*=0.52, *R*²=0.27, mean squared error=1.17, *F*_3, 332_=40.80; *P*<.001.

## Discussion

### Principal Findings

**Figure 4:**
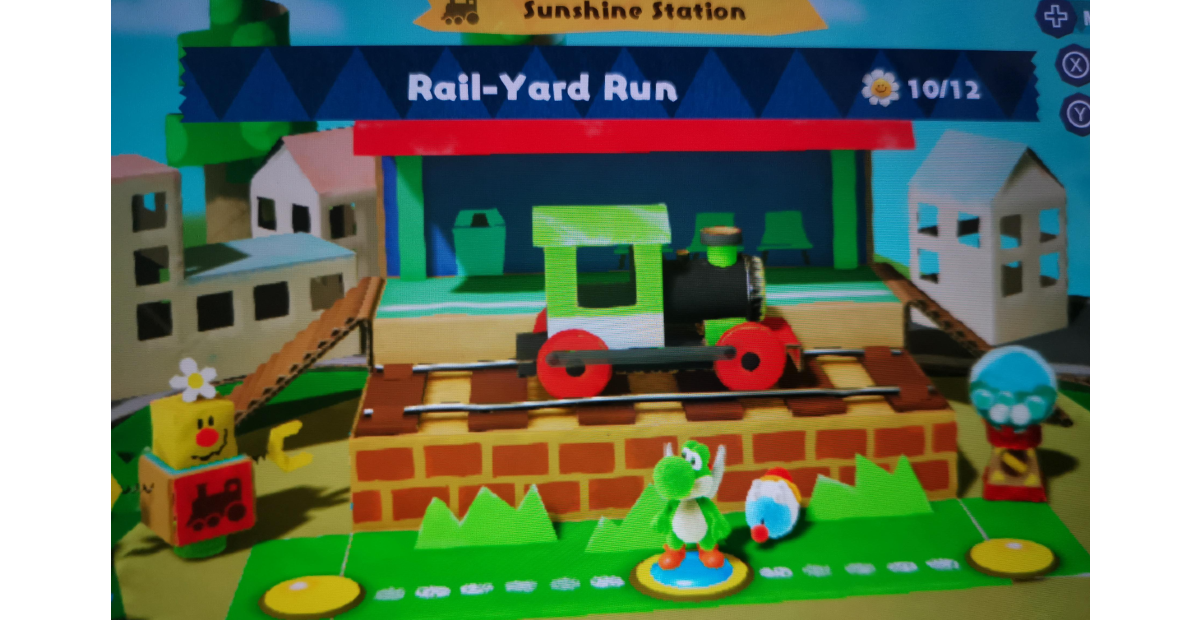
Joyous scenery in *Yoshi’s Crafted World* (reproduced with permission from Nintendo).

The findings of this study show that players appreciated Super Mario Bros. and Yoshi games’ affordance of childlike wonder as well as enhanced feelings of happiness. In addition, the findings of the study indicated the extent to which players noted Super Mario Bros. and Yoshi games’ ability to reduce burnout risks. The findings of the quantitative study further showed that Super Mario Bros. and Yoshi games’ affordance of childlike wonder was positively associated with people’s overall happiness in life. Finally, happiness was negatively associated with burnout risk of study respondents, hence indicating the potential for popular video games such as Super Mario Bros. and Yoshi games to help reduce burnout risk. The colorful, low-pressure worlds of Yoshi games were described by interviewees as a “vacation for the mind.” Figure 4 illustrates this atmosphere, conveying the playful and uplifting tone that helps transform ordinary play into a restorative emotional experience.

### Comparison to Prior Work

Prior work has noted the role video games can play in people’s lives, including enhanced cognitive escapism and relaxation [28-30]. Beyond escapism, prior research using Self-Determination Theory indicates that games can support basic psychological needs for autonomy, competence, and relatedness, which are associated with intrinsic motivation and need-contingent, short-term improvements in well-being [55]. Players have also been noted to engage with games strategically for mood management and coping-based emotion regulation, selecting particular gameplay challenges or mechanics to reduce stress, elevate mood, or regain a sense of control [56-58]. How popular video games such as Super Mario Bros. and Yoshi may affect young adults’ ability to look at the world and their lives in renewed, childlike wonder and how this may affect their overall happiness and reduce risk of burnout, however, remains unanswered. Moreover, nostalgia has been noted to promote well-being by drawing on cherished past experiences to restore positive affect, meaning, and a sense of connection [59], and mainstream, long-running franchises often cue players’ nostalgia, especially when they prompt recall of past play experience with familiar series, settings, or characters [60]. However, nostalgia alone may not fully explain the uplifting, playful stance reported by players of whimsical franchises such as Super Mario and Yoshi, pointing to a distinct experiential state-childlike wonder-that has not yet been systematically examined.

While prior research examined how various gamification techniques may enhance people’s feelings of hope or connection with a new digital offering [61-64], critical questions remained about the role highly popular video games, such as Super Mario Bros. and Yoshi, may play in reducing people’s burnout risk. Although previous studies shed light on designing offerings that make people’s lives easier, put a smile on their face, and possibly inspire them and help form a stronger bond with them [65-70], work has only recently begun to examine how specific content and structural features of games shape emotional outcomes. For example, game aesthetics such as visual style, world design, music, and pacing have been proposed to elicit both hedonic enjoyment and deeper, reflective emotions [71,72]. Likewise, soundtrack music in games has been shown to shape players’ emotional responses, for example, by intensifying positive emotions and enhancing horror experiences [73]. In addition, in-game success has been found to predict mood repair and satisfaction of competence and autonomy to mediate effects on enjoyment [74]. Notably, these mechanisms have seldom been linked to broader indicators of well-being, such as sustained happiness or resilience to burnout, particularly among young adults.

Recent reviews suggest that commercial video games show promise for alleviating symptoms of anxiety and depression [2,75]. While these findings are encouraging, the present study takes a different perspective: rather than positioning entertainment games as treatments, it considers whether everyday play can function as a subtle, restorative micro-environment. Thus, the current study contributes to work examining the influence of gaming on mental well-being and, in doing so, further helps bridge gaming and mental health science [76,77] by identifying childlike wonder as a unique emotional pathway through which mainstream games may support happiness and reduce burnout risk.

### Limitations

The benefits of video gameplay, including Super Mario Bros. and Yoshi, are likely to be contingent on moderate, voluntary play primarily for enjoyment. Compulsive video gameplay or excessive escapism used solely to avoid real problems can be maladaptive and undermine optimism by neglecting real-world agency. Moreover, playing Super Mario and Yoshi games can help mitigate individual experiences of burnout, but does not address systemic causes (eg, excessive workload, unfair practices). Thus, it may complement, not replace, organizational interventions. Moreover, in this study, we examined self-reported burnout risk. Future research examining the long-term effects of gameplay on actual burnout risk is richly deserving. In addition, we encourage future work to study the potential effects of gameplay on people of varying ages, demographic backgrounds, etc., and examine the influence that such gameplay may have on various aspects of people’s overall well-being. Examining such variations could reveal how developmental context and lived experience shape how popular games relate to well-being.

### Future Research

The specific design elements of Super Mario Bros., as well as Yoshi games and their focus on skill-based mastery, low violence, predictable rules, achievable goals, and positive aesthetics, may be crucial for fostering the beneficial aspects described in this study. Not all video games offer this combination. Future research that further investigates the potential positive and negative effects of video gameplay on human happiness, resilience, and empathy is richly deserving. Current work examines the extent to which internet use may affect people’s anxiety and stress [78,79]; however, there is also work suggesting offerings to put a smile on users’ faces and make their lives easier [80-84].

Because additional interviews continued to offer new and nuanced insights, saturation in our qualitative study may not have been achieved, and we invite future research to explore and further investigate the role of popular games in people’s lives and their potential impact on the resilience, coping ability, physical and emotional health, as well as overall well-being of people across ages and various lifestyles. As previous research indicates an increasing number of people searching for meaning in their lives and feeling detached from nature as well as fellow human beings [85-88], additional work studying the effects of popular video games on people’s empathy, perceived loneliness, biophilia, as well as gratitude and levels of overconsumption is richly deserving.

Nintendo’s Super Mario Bros., as well as Yoshi games, can be more than just platformers. They are carefully constructed wonder simulators. They reject the high-stakes, high-stress models of many modern games in favor of an experience that prioritizes safety, curiosity, and joy. For a person whose life may be dominated by deadlines, overwhelming demands, and a perceived loss of control, stepping into Super Mario’s or Yoshi’s crafted world can be a form of digital therapy. It allows the person to practice being curious again, to experience uncomplicated joy, to succeed in small, measurable ways, and to simply exist in a moment of bright, colorful, and kind-hearted joy.

These games, in essence, are a vacation for the mind, facilitating the very childlike wonder that burnout so effectively extinguishes. Intentional game design can do more than just entertain - it can heal, restore, and inspire. Future research further exploring the various ways gameplay may affect people’s outlook and approach to life is richly deserving.

### Conclusions

Insights from the qualitative study indicated players’ appreciation of Super Mario and Yoshi games’ affordance of childlike wonder, happiness in life, and a reduction in burnout risk. In addition to this, the findings of the quantitative analyses showed how Super Mario and Yoshi games’ affordance of childlike wonder influenced happiness in life and significantly reduced burnout risk. These video games, hence, can be effective vehicles for enhanced mental well-being. More specifically, the current findings show that Super Mario Bros. and Yoshi games act as an effective antidote by creating a virtual space that is the psychological inverse of a burnout-inducing environment.

In conclusion, the findings for this study demonstrate that popular video games, such as Super Mario Bros. and Yoshi, can enhance childlike wonder, which in turn increases overall happiness and reduces burnout risk. This work is innovative in identifying childlike wonder as a previously underexplored emotional mechanism that links everyday gameplay with improved well-being. It differs from prior studies that have focused mainly on escapism, stress relief, or need satisfaction by showing how mainstream entertainment games can evoke curiosity, playfulness, and joy that counteract cynicism and exhaustion. The study contributes to the growing field connecting gaming and mental health by positioning popular titles as restorative digital spaces rather than merely sources of distraction. Practically, the findings suggest that thoughtfully designed, low-pressure games may serve as simple, real-world tools to rekindle curiosity, uplift mood, and support resilience among young adults facing burnout.

## Appendix

Multimedia Appendix 1 STROBE checklist for cross-sectional studies.

## Abbreviations

AMOS: Analysis of Moment Structures
BootSE: bootstrapped standard error
MSE: mean squared error
RA: research assistant
SPSS: Statistical Package for the Social Science
STROBE: Strengthening the Reporting of Observational Studies in Epidemiology

## Data Availability

The data for the study are available from the authors on reasonable request.
